# YOLOv5s-Based Image Identification of Stripe Rust and Leaf Rust on Wheat at Different Growth Stages

**DOI:** 10.3390/plants13202835

**Published:** 2024-10-10

**Authors:** Qian Jiang, Hongli Wang, Zhenyu Sun, Shiqin Cao, Haiguang Wang

**Affiliations:** 1 College of Plant Protection, China Agricultural University, Beijing 100193, China; 18188292276@163.com (Q.J.); wanghli12@163.com (H.W.); 2 Institute of Plant Protection, Gansu Academy of Agricultural Sciences, Lanzhou 730070, China; szy20020815@163.com (Z.S.); caoshiqin6702@163.com (S.C.)

**Keywords:** wheat stripe rust, wheat leaf rust, disease identification, image recognition, growth stage

## Abstract

Stripe rust caused by *Puccinia striiformis* f. sp. *tritici* and leaf rust caused by *Puccinia triticina*, are two devastating diseases on wheat, which seriously affect the production safety of wheat. Timely detection and identification of the two diseases are essential for taking effective disease management measures to reduce wheat yield losses. To realize the accurate identification of wheat stripe rust and wheat leaf rust during the different growth stages, in this study, the image-based identification of wheat stripe rust and wheat leaf rust during different growth stages was investigated based on deep learning using image processing technology. Based on the YOLOv5s model, we built identification models of wheat stripe rust and wheat leaf rust during the seedling stage, stem elongation stage, booting stage, inflorescence emergence stage, anthesis stage, milk development stage, and all the growth stages. The models were tested on the different testing sets in the different individual growth stages and in all the growth stages. The results showed that the models performed differently in disease image identification. The model based on the disease images acquired during an individual growth stage was not suitable for the identification of the disease images acquired during the other individual growth stages, except for the model based on the disease images acquired during the milk development stage, which had acceptable identification performance on the testing sets in the anthesis stage and the milk development stage. In addition, the results demonstrated that wheat growth stages had a great influence on the image identification of the two diseases. The model built based on the disease images acquired in all the growth stages produced acceptable identification results. Mean F1 Score values between 64.06% and 79.98% and mean average precision (mAP) values between 66.55% and 82.80% were achieved on each testing set composed of the disease images acquired during an individual growth stage and on the testing set composed of the disease images acquired during all the growth stages. This study provides a basis for the image-based identification of wheat stripe rust and wheat leaf rust during the different growth stages, and it provides a reference for the accurate identification of other plant diseases.

## 1. Introduction

Stripe rust caused by *Puccinia striiformis* f. sp. *tritici* (*Pst*) and leaf rust caused by *Puccinia triticina* (*Pt*), two important airborne fungal diseases on wheat (*Triticum aestivum*), occur widely in wheat-growing areas worldwide and can cause severe yield losses of wheat [1,2,3]. Both *Pst* and *Pt* are heteroecious and can produce five kinds of spores, including pycniospore, aeciospore, urediospore, teliospore, and basidiospore, throughout their life cycles [4,5]. Stripe rust and leaf rust can occur during all growth stages of wheat. In the seedling growth stage, the symptoms of stripe rust are easily confused with those of leaf rust because both diseases induce scattered uredia on the wheat leaves [6]. In the adult plant stage, the uredia produced by *Pst* infection are usually distributed in intermittent, long, and narrow stripes on the wheat leaves [6,7], while the uredia produced by *Pt* infection are irregularly arranged on the leaves. In different growth stages, generally, the disease resistance levels of wheat are inconsistent, and the disease symptoms are different to a certain extent. In the field, the traditional identification method of wheat stripe rust and wheat leaf rust is mainly carried out by manual visual observation. However, many experienced plant protection personnel or technicians do not have enough time to reach the field to diagnose wheat diseases and provide services for farmers, which cannot meet the requirements for real-time disease diagnosis and monitoring. Rapid and accurate diagnosis of wheat stripe rust and wheat leaf rust is the key to the prevention and control of the diseases.

With the rapid development of machine learning, the identification and monitoring of plant diseases based on information technology have received extensive attention [8]. Techniques for image acquisition and image processing have rapidly developed, and the corresponding technological progress has turned plant disease image processing issues (including segmentation, feature extraction, and identification of plant disease images) into hot research topics [9,10,11,12,13,14]. Many studies on plant disease image identification using traditional image processing technology have been reported [9,10,11,12,14,15]. Using traditional image processing technology, the disease lesions are segmented from plant disease images, then the useful features are extracted from the disease lesion images and are selected for modeling. Finally, machine learning methods are used to implement the identification of the plant disease images. Studies on image identification of wheat stripe rust and wheat leaf rust using traditional image processing technology have also been reported [16,17,18]. The performance of traditional image processing technology in identifying plant disease images is affected by various factors, including the number, quality, and representativeness of the acquired disease images; the image processing techniques and methods used; the extracted disease image features and feature selection methods; and the machine learning methods used. In the traditional identification methods of plant disease images, the feature values, position distribution, and area sizes of the target regions in the disease images can change with the different development stages of plant diseases [9,11,18]. Furthermore, if the disease images are acquired from the same kind of plant or if the number of the acquired disease images is not enough, the identification performance and generalization ability of plant disease identification models can be affected [11,19]. Deep learning can be used to realize the automatic extraction of image features and pattern recognition, and it shows outstanding performance in image recognition, image segmentation, target detection, and other fields, and thus the amount of research on deep learning applied to plant disease identification and plant disease segmentation is increasing [13,14,20,21,22,23,24,25,26,27]. At present, some deep learning models have been applied to image identification of wheat stripe rust and wheat leaf rust [28,29,30]. Lu et al. [28] implemented the identification and localization of wheat diseases (including powdery mildew, smut, black chaff, stripe rust, leaf blotch, and leaf rust) based on an in-field wheat disease image data set using deep multiple instance learning (DMIL) under two fully convolutional network (FCN) architectures (VGG-FCN-VD16 and VGG-FCN-S), achieving mean recognition accuracies of 97.95% and 95.12%, respectively. Based on the images of wheat powdery mildew, wheat stripe rust, and wheat leaf rust acquired in in-field environments, Feng et al. [29] developed a wheat leaf disease image recognition model using the lightweight convolutional neural network MobileNetV2 and transfer learning under the deep learning framework TensorFlow 2.0, achieving an average accuracy of 99.96% for image recognition of the three wheat diseases. Jiang et al. [30] conducted a comparison of seven convolutional neural networks (CNN) (including VGG-16, Inception-v3, ResNet-50, DenseNet-121, EfficentNet-B6, ShuffleNet-v2, and MobileNetV3) for the identification of field images of powdery mildew, leaf rust, and stripe rust on wheat.

Redmon et al. [31] proposed a target detection model named YOLO (You Only Look Once). YOLO treats target detection in an image as a regression problem, taking the image as input and outputting the locations and classes of the targets. Compared with traditional target detection models, YOLO has the advantages of rapid running speed, detection in a global image, learning the generalized feature representation of the target, etc. YOLOv5 is one of the YOLO series models, which includes four network models: YOLOv5s, YOLOv5m, YOLOv5l, and YOLOv5x. Among them, YOLOv5s is the one with the smallest depth and the smallest feature map width. YOLOv5 has been applied to image-based plant disease identification [32,33,34,35]. Mathew and Mahesh [32] trained a YOLOv5-based model with collected images of bell peppers and achieved a good performance of the trained model in detecting bacterial spot in leaf images of bell peppers. Qiu et al. [33] developed a YOLOv5l-based method and an application (app) named ‘HLBdetector’ for image-based detection of citrus Huanglongbing (HLB). Xu et al. [34] developed a lightweight ShuffleNetv2-based YOLOv5s network model for the detection of wheat stripe rust, achieving better detection performance and faster detection speed. A lightweight YOLO-V5s-based model for apple leaf disease detection (ALAD-YOLO) was developed by Xu and Wang [35] and a detection accuracy of 90.2% was achieved in the apple leaf disease detection experiment using ALAD-YOLO. As far as we know, YOLOv5 has not been applied to the identification of wheat stripe rust and wheat leaf rust.

In recent years, some advances have been made in research on image identification of wheat stripe rust and wheat leaf rust, but there are still various challenges and difficulties to be faced. At the present time, public image data sets of wheat stripe rust and wheat leaf rust are still scarce. In most of the existing studies, the investigations of image identification of the two wheat diseases were carried out based on the disease images with simple backgrounds from self-built image data sets, rather than based on ones acquired in complex field environments. Image features of plant diseases can be affected by many factors, such as plant variety, disease development stage, and image acquisition environment [9,11,18,24]. During image identification of wheat stripe rust and wheat leaf rust, identification performance can be affected by changes in disease image features that may result from changes in disease development stages. Most of the existing studies on image-based plant disease identification are based on images acquired in a single plant growth stage or images acquired in unclear plant growth stages, and there is a lack of research on algorithms or models for identifying disease images acquired during different plant growth stages. To our best knowledge, there has been no reported research on the differences between the performances of the built models in identifying disease images acquired in different growth stages of plants. Building image identification models of stripe rust and leaf rust on wheat in different growth stages needs further research. Therefore, in this study, data sets of disease images of stripe rust and leaf rust on wheat with different backgrounds acquired at the different growth stages were constructed, and then effective methods for image identification of the two diseases on wheat in different growth stages were investigated.

To overcome the influence of wheat growth stages on disease image identification, image-based identification of stripe rust and leaf rust on wheat in the different growth stages was investigated using deep learning in this study. The images of wheat stripe rust and wheat leaf rust with complex backgrounds and single backgrounds were acquired at the seedling growth stage, stem elongation stage, booting stage, inflorescence emergence stage, anthesis stage, and milk development stage of wheat (in this study, the six growth stages refer to the stages of Z12–Z14, Z30–Z39, Z40–Z49, Z50–Z59, Z60–Z69, and Z70–Z77, respectively, according to the decimal code for growth stages of cereals determined by Zadoks et al. [36]) in an indoor environment and in the field using a digital camera and a smartphone. Then, a total of seven disease image data sets of the seedling growth stage, stem elongation stage, booting stage, inflorescence emergence stage, anthesis stage, milk development stage, and all the growth stages were constructed. Based on the YOLOv5s model and the constructed image data sets, the image-based identification models of wheat stripe rust and wheat leaf rust at the seedling stage, stem elongation stage, booting stage, inflorescence emergence stage, anthesis stage, milk development stage, and all the growth stages were trained, validated, and tested. The main aims of this study are to investigate whether there are differences in the identification of the images of stripe rust and leaf rust on wheat at the different growth stages based on the YOLOv5s model and to break the limitations of unsatisfactory disease identification performance caused by different disease image features extracted during different growth stages, which can provide a reference for further extracting disease image features and building disease image identification models. A reference can be provided for accurately identifying the two wheat diseases, which is conducive to timely scientific disease control measures that reduce yield losses in wheat.

## 2. Results

### 2.1. Identification Results of the Disease Image Identification Models Based on the Training Sets in the Individual Growth Stages

For the YOLOv5s-based disease image identification models based on the training sets composed of the disease images acquired at the individual growth stages (the seedling stage, stem elongation stage, booting stage, inflorescence emergence stage, anthesis stage, and milk development stage), the identification results of the testing sets composed of the disease images acquired during the different growth stages are shown in Table 1, Table 2, Table 3, Table 4, Table 5 and Table 6.

#### 2.1.1. Identification Results of the Disease Image Identification Model Based on the Training Set in the Seedling Stage

Using the YOLOv5s-based disease image identification model based on the training set in the seedling stage, the disease images in the testing sets in the different individual growth stages were identified, and the results, as shown in Table 1, indicated that there were great differences between the performances of the identification model on the different testing sets. The mean F1 Score and mean average Precision (mAP) for the image identification of the testing set in the seedling stage were the highest, with values of 78.12% and 82.45%, respectively. The mAP for the image identification of the testing set in the inflorescence emergence stage was the lowest, with a value of 28.20%. The Precision, Recall, F1 Score, and Average Precision (AP) for the identification of the images of wheat stripe rust in the testing set in the seedling stage were the highest, with values of 81.30%, 85.80%, 83.49%, and 89.60%, respectively. The Precision for the identification of the images of wheat stripe rust in the testing set in the anthesis stage was the lowest, with a value of 34.00%. Both the Recall and F1 Score for the identification of the images of wheat stripe rust in the testing set in the stem elongation stage were the lowest, with values of 10.20% and 17.32%, respectively. The AP for the identification of the images of wheat stripe rust in the testing set in the milk development stage was the lowest, with a value of 19.83%. For the identification of the images of wheat leaf rust, the Precision, F1 Score, and AP of the testing set in the seedling stage were the highest, with values of 76.20%, 72.75%, and 75.30%, respectively; the Recall of the testing set in the anthesis stage was the highest, with a value of 70.60%; and the Precision, Recall, F1 Score, and AP of the testing set in the inflorescence emergence stage were the lowest, with values of 21.10%, 44.30%, 28.59%, and 18.90%, respectively. The results demonstrated that, for the YOLOv5s-based image identification model based on the training set of wheat stripe rust and wheat leaf rust in the seedling stage, the identification performance on the testing set in the seedling stage (the same growth stage at which the images in the training set used for modeling were acquired) was the best, and the performance on the testing set in the inflorescence emergence stage was the worst.

#### 2.1.2. Identification Results of the Disease Image Identification Model Based on the Training Set in the Stem Elongation Stage

As shown in Table 2, when using the YOLOv5s-based disease image identification model based on the training set in the stem elongation stage, on the whole, the identification performance on the testing sets in the different individual growth stages was not very good, among which there were relatively great differences. The mean F1 Score and the mAP for the image identification of the testing set in the stem elongation stage were the highest, with values of 63.08% and 63.55%, respectively. Both the mean F1 Score and the mAP for the image identification of the testing set in the milk development stage were the lowest, with values of 34.09% and 27.75%, respectively. For the identification of the images of wheat stripe rust, the Precision, F1 Score, and AP of the testing set in the stem elongation stage were the highest, with values of 68.80%, 61.38%, and 63.20%, respectively; the Recall of the testing set in the seedling stage was the highest, with a value of 57.80%; and the Precision, Recall, F1 Score, and AP of the testing set in the milk development stage were the lowest, with values of 21.90%, 26.00%, 23.77%, and 15.70%, respectively. For identification of the images of wheat leaf rust, the Precision, Recall, F1 Score, and AP of the testing set in the stem elongation stage were the highest, with values of 63.80%, 65.80%, 64.78%, and 63.90%, respectively; and the Precision, Recall, F1 Score, and AP of the testing set in the inflorescence emergence stage were the lowest, with values of 33.60%, 38.80%, 36.01%, and 27.00%, respectively. The results demonstrated that, for the YOLOv5s-based image identification model based on the training set of wheat stripe rust and wheat leaf rust in the stem elongation stage, the identification performance on the testing set in the stem elongation stage (the same growth stage at which the images in the training set used for modeling were acquired) was the best, and the performance on the testing set in the milk development stage was the worst.

#### 2.1.3. Identification Results of the Disease Image Identification Model Based on the Training Set in the Booting Stage

Using the YOLOv5s-based disease image identification model based on the training set in the booting stage, the disease images in the testing sets in the individual different growth stages were identified. As shown in Table 3, on the whole, the identification performance of the model was not very good, and the differences between the identification performances on the testing sets in the different growth stages were relatively great. The mean F1 Score and the mAP for the image identification of the testing set in the booting stage were the highest, with values of 62.45% and 64.85%, respectively. The mean F1 Score for the image identification of the testing set in the stem elongation stage was the lowest, with a value of 49.39%. The mAP for the image identification of the testing set in the milk development stage was the lowest, with a value of 47.85%. For the identification of the images of wheat stripe rust, the Precision of the testing set in the inflorescence emergence stage was the highest, with a value of 77.70%; the Recall, F1 Score, and AP of the testing set in the booting stage were the highest, with values of 65.20%, 66.95%, and 72.60%, respectively; the Precision and AP of the testing set in the milk development stage were the lowest, with values of 46.60% and 46.80%, respectively; and the Recall and F1 Score of the testing set in the stem elongation stage were the lowest, with values of 29.00% and 41.86%, respectively. For the identification of the images of wheat leaf rust, the Precision of the testing set in the stem elongation stage was the highest, with a value of 63.30%; the Recall and F1 Score of the testing set in the anthesis stage were the highest, with values of 64.60% and 58.05%, respectively; the AP of the testing set in the booting stage was the highest, with a value of 57.10%; the Precision of the testing set in the anthesis stage was the lowest, with a value of 52.70%; and the Recall, F1 Score, and AP of the testing set in the seedling stage were the lowest, with values of 44.00%, 48.37%, and 45.10%, respectively. The results demonstrated that, for the YOLOv5s-based image identification model based on the training set of wheat stripe rust and wheat leaf rust in the booting stage, the identification performance on the testing set in the booting stage (the same growth stage at which the images in the training set used for modeling were acquired) was the best, and the identification performances on the testing sets in the stem elongation stage and the milk development stage were the worst.

#### 2.1.4. Identification Results of the Disease Image Identification Model Based on the Training Set in the Inflorescence Emergence Stage

Using the YOLOv5s-based disease image identification model based on the training set in the inflorescence emergence stage, the disease images in the testing sets in the different individual growth stages were identified, and the results are shown in Table 4. The results showed that there were relatively great differences between the identification performances of the built model on the testing sets in the different growth stages. The mean F1 Score and the mAP for the image identification of the testing set in the inflorescence emergence stage were the highest, with values of 69.06% and 71.45%, respectively. The mean F1 Score for the image identification of the testing set in the stem elongation stage was the lowest, with a value of 39.39%. The mAP for the image identification of the testing set in the seedling stage was the lowest, with a value of 43.10%. The Precision, Recall, F1 Score, and AP for the identification of the images of wheat stripe rust in the testing set in the inflorescence emergence stage were the highest, with values of 74.70%, 75.90%, 75.30%, and 79.90%, respectively. The Precision for the identification of the images of wheat stripe rust in the testing set in the seedling stage was the lowest, with a value of 53.40%. The Recall, F1 Score, and AP for the identification of the images of wheat stripe rust in the testing set in the stem elongation stage were the lowest, with values of 20.30%, 30.07%, and 38.90%, respectively. The Precision, Recall, F1 Score, and AP for the identification of the images of wheat leaf rust in the testing set in the inflorescence emergence stage were the highest, with values of 67.30%, 58.90%, 62.82%, and 63.00%, respectively. The Precision, Recall, F1 Score, and AP for the identification of the images of wheat stripe rust in the testing set in the seedling stage were the lowest, with values of 49.40%, 32.10%, 38.91%, and 33.00%, respectively. The results demonstrated that, for the YOLOv5s-based image identification model based on the training set of wheat stripe rust and wheat leaf rust in the inflorescence emergence stage, the identification performance on the testing set in the inflorescence emergence stage (the same growth stage at which the images in the training set used for modeling were acquired) was the best, and the identification performances on the testing sets in the seedling stage and the stem elongation stage were the worst.

#### 2.1.5. Identification Results of the Disease Image Identification Model Based on the Training Set in the Anthesis Stage

Using the YOLOv5s-based disease image identification model based on the training set in the anthesis stage, the disease images in the testing sets in the different individual growth stages were identified, and the results are shown in Table 5. There were great differences between the identification performances of the built model on the testing sets in the different growth stages of wheat. The mean F1 Score and the mAP for the image identification of the testing set in the anthesis stage were the highest, with values of 72.11% and 75.30%, respectively. The mean F1 Score for the image identification of the testing set in the stem elongation stage was the lowest, with a value of 31.19%. The mAP for the image identification of the testing set in the seedling stage was the lowest, with a value of 41.50%. For the identification of the images of wheat stripe rust, the Precision of the testing set in the inflorescence emergence stage was the highest, with a value of 76.80%; the Recall, F1 Score, and AP of the testing set in the anthesis stage were the highest, with values of 71.80%, 70.11%, and 72.70%, respectively; the Precision of the testing set in the milk development stage was the lowest, with a value of 64.40%; the Recall, F1 Score, and AP of the testing set in the stem elongation stage were the lowest, with values of 14.50%, 24.32%, and 44.40%, respectively. For the identification of the images of wheat leaf rust, the Precision, Recall, F1 Score, and AP of the testing set in the anthesis stage were the highest, with values of 71.60%, 76.80%, 74.11%, and 77.90%, respectively; the Precision, Recall, F1 Score, and AP of the testing set in the seedling stage were the lowest, with values of 44.40%, 27.40%, 33.89%, and 28.40%, respectively. The results demonstrated that, for the YOLOv5s-based image identification model based on the training set of wheat stripe rust and wheat leaf rust in the anthesis stage, the identification performance on the testing set in the anthesis stage (the same growth stage at which the images in the training set used for modeling were acquired) was the best, and the identification performances on the testing sets in the seedling stage and the stem elongation stage were the worst.

#### 2.1.6. Identification Results of the Disease Image Identification Model Based on the Training Set in the Milk Development Stage

Using the YOLOv5s-based disease image identification model based on the training set in the milk development stage, the disease images in the testing sets in the different individual growth stages were identified, and the results, as shown in Table 6, indicated that there were relatively great differences between the performances of the built identification model on the different testing sets. The mean F1 Score for the image identification of the testing set in the anthesis stage was the highest, with a value of 65.29%. The mAP for the image identification of the testing set in the milk development stage was the highest, with a value of 67.20%. The mean F1 Score for the image identification of the testing set in the stem elongation stage was the lowest, with a value of 29.75%. The mAP for the image identification of the testing set in the seedling stage was the lowest, with a value of 35.60%. For the identification of the images of wheat stripe rust, the Precision of the testing set in the inflorescence emergence stage was the highest, with a value of 64.30%; the Recall, F1 Score, and AP of the testing set in the milk development stage were the highest, with values of 65.10%, 64.49%, and 68.00%, respectively; the Precision of the testing set in the seedling stage was the lowest, with a value of 49.20%; and the Recall, F1 Score, and AP of the testing set in the stem elongation stage were the lowest, with values of 11.10%, 18.33%, and 32.30%, respectively. For the identification of the images of wheat leaf rust, the Precision, Recall, F1 Score, and AP of the testing set in the anthesis stage were the highest, with values of 66.50%, 68.70%, 67.58%, and 68.60%, respectively; the Recall of the testing set in the milk development stage was also the highest, with a value of 68.70%; values of the Precision, F1 Score, and AP of the testing set in the milk development stage were 62.60%, 65.51%, and 66.40%, respectively; the Precision of the testing set in the inflorescence emergence stage was the lowest, with a value of 42.70%; and the Recall, F1 Score, and AP of the testing set in the seedling stage were the lowest, with values of 25.20%, 32.91%, and 29.90%, respectively. The results demonstrated that, for the YOLOv5s-based image identification model based on the training set of wheat stripe rust and wheat leaf rust in the milk development stage, the identification performances on the testing sets in the anthesis stage and the milk development stage were the best, and the identification performances on the testing sets in the seedling stage and the stem elongation stage were the worst.

### 2.2. Identification Results of the Disease Image Identification Model Based on the Training Set at All the Growth Stages

Using the YOLOv5s-based disease image identification model based on the training set at all the growth stages, the disease images in the testing sets in the different individual growth stages and all the growth stages were identified, and the results are shown in Table 7. The results showed that the mean F1 Score and mAP for the image identification of the testing set in the seedling stage were the highest, with values of 79.98% and 82.80%, and the mean F1 Score and mAP for the image identification of the testing set in the stem elongation stage were the lowest, with values of 64.06% and 66.55%. For the identification of the images of wheat stripe rust, the Precision, Recall, F1 Score, and AP of the testing set in the seedling stage were the highest, with values of 84.40%, 84.40%, 84.40%, and 89.00%, respectively; the Precisions of the testing sets in the anthesis stage and milk development stage were equal and the lowest, with a value of 69.00%; and the Recall, F1 Score, and AP of the testing set in the stem elongation stage were the lowest, with values of 56.80%, 62.71%, and 66.80%, respectively. For the identification of the images of wheat leaf rust, the Precision, Recall, F1 Score, and AP of the testing set in the seedling stage were the highest, with values of 75.60%, 75.50%, 75.55%, and 76.60%, respectively; the Precision, Recall, F1 Score, and AP of the testing set in the booting stage were the lowest, with values of 61.10%, 59.00%, 60.03%, and 61.60%, respectively. The results demonstrated that, for the YOLOv5s-based image identification model based on the training set of wheat stripe rust and wheat leaf rust at all the growth stages, the identification performances on the images of wheat stripe rust and wheat leaf rust, the images of the individual disease caused by *Pst* (i.e., wheat stripe rust), and the images of the individual disease caused by *Pt* (i.e., wheat leaf rust) in the testing set in the seedling stage were the best; the identification performances on the images of wheat stripe rust and wheat leaf rust and the images of the individual disease caused by *Pst* (i.e., wheat stripe rust) in the testing set in the stem elongation stage were the worst; and the identification performance on the images of the individual disease caused by *Pt* (i.e., wheat leaf rust) in the testing set in the booting stage was the worst.

The above results demonstrated that, for the YOLOv5s-based image identification models based on the training sets of wheat stripe rust and wheat leaf rust in the individual growth stages, the best identification performances were achieved on the testing sets in the same individual growth stages; for the YOLOv5s-based image identification model based on the training set of wheat stripe rust and wheat leaf rust in all the growth stages, the identification performances achieved on the testing sets in the seedling stage, inflorescence emergence stage, anthesis stage, all the growth stages, milk development stage, booting stage, and stem elongation stage ranked from high to low, and were acceptable.

## 3. Discussion

Image acquisition is the basis of plant disease image identification. With the growth of wheat plants and the change in the growth stages of wheat, the leaf cuticle will gradually thicken, the leaves may be chlorotic and yellowing in the later stages, and the color and shapes of uredia of wheat stripe rust and wheat leaf rust may be different at different periods, which will affect the extracted disease image features, thus further affecting the disease image identification. The changes in illumination conditions may lead to the difference between the color obtained from a disease image and the color obtained from human visual perception, thus affecting the extracted disease image features. At present, the images in the data sets used for image identification of wheat stripe rust and wheat leaf rust were acquired at an individual growth stage or not classified according to the growth stages [16,17,18,28,29,30]. Moreover, most of the disease images used had simple backgrounds and a single disease location, and they were acquired under similar illumination conditions. However, in actual agricultural production, wheat can be infected with *Pst* and *Pt* in the different growth stages, and different severity levels of wheat stripe rust and wheat leaf rust can occur at diverse disease locations under different environments. Therefore, to adapt to the actual agricultural production, the acquired disease images should contain complex backgrounds in the field, and factors such as image acquisition equipment and illumination conditions should be taken into account when acquiring disease images. Thus, the constructed disease image data sets may contain more abundant images, providing a basis for obtaining trained disease image identification models with greater robustness. Generally, there is a process of continuous expansion and change in the lesions during disease lesion formation, so disease image identification in the different development stages of plant diseases still needs further research [37]. Until now, there have been no reported studies on image identification of plant diseases in the different growth stages. Therefore, it is necessary to carry out related research on the image identification of plant diseases in the different growth stages to investigate whether the disease image features extracted at an individual growth stage are also applicable to the identification of the same kind of disease in other growth stages, which provides a new idea for the construction of image data sets for disease identification using image processing technology. The results of this study showed that, during the process of building a disease image identification model, factors such as the differences between disease image features in the different growth stages should be taken into account. When selecting disease images for model training, disease images in the different growth stages should be included, rather than only disease images acquired in an individual growth stage or a minority of individual growth stages. Disease image identification modeling should be conducted on the basis of fully considering the different characteristics of disease images in the different growth stages, which may provide a solution to improve the identification performance of the disease identification models.

When using target detection models for plant disease image identification, image labeling and model optimization have a great influence on model building and identification performance of the built models. Manual labeling of disease images is time-consuming and labor-intensive, and the subjectivity of labeling affects the results of labeling [24]. CNN-based semi-supervised learning algorithms and unsupervised learning algorithms can reduce the cost and time of data labeling, and they have been used in many fields of plant phenotype research [38]. In this study, for wheat leaf rust, the produced uredia were scattered on the wheat leaves, and there were multiple uredia on a single wheat leaf, which brought great challenges to the image labeling, and this may affect the training of the YOLOv5s-based image identification models using the training sets, resulting in relatively low values in the evaluation metrics for identification of wheat leaf rust using the built models. In further studies, the results of disease image labeling can be checked, or the images can be re-labeled, the hyperparameters of the YOLOv5s model can be adjusted, and the model can be re-trained to build more optimized image identification models for image identification of wheat stripe rust and wheat leaf rust in the different growth stages.

In this study, although acceptable disease identification results were obtained by building a disease image identification model based on the training set composed of the images acquired in all the growth stages, the identification performance of the model was not particularly good. By increasing the number of disease images acquired at each individual growth stage, the disease image data set may contain more disease images acquired under complex environments so as to improve the identification performance and generalization ability of the built model. On the other hand, the model can be more optimized by changing the model structure, resulting in improved identification performance. Based on the original deep learning models, good identification performance may not be achieved using transfer learning. The original deep learning models can be improved through modification with other network structures and mechanisms, and the disease image identification models based on modified deep learning architectures may be more suitable for target detection and disease identification. For instance, Ma et al. [14] used YOLOv8 as the base model, replaced the backbone network of YOLOv8 with a PP-LCNet (PP-lightweight CPU network), introduced the depthwise separable convolution (DepthSepConv) structure into the backbone layer, and then added a global attention mechanism (GAM) module and a lightweight content-aware reassembly of features (CARAFE) module to the neck section. They subsequently replaced the original loss function with the Wise-IoU (weighted interpolation of sequential evidence for intersection over union) boundary loss function, and finally, they built a lightweight detection model PGCW-YOLOv8 for wheat diseases. Using this PGCW-YOLOv8 model to identify images of wheat leaf rust, wheat stripe rust, wheat scab, wheat glume blotch, wheat powdery mildew, healthy wheat ears, and healthy wheat leaves, much better identification performance was achieved.

To facilitate practical applications, a number of PC-based computer systems and apps for plant disease image identification have been developed [13,28,33,39,40]. Based on the color and shape features extracted from the images of common diseases of wheat, Wang et al. [39] developed an identification system of common wheat leaf diseases using the VC++ platform in combination with image processing technology, achieving an identification accuracy of more than 96% in the image identification of wheat powdery mildew, wheat leaf rust, wheat stripe rust, and wheat stem rust using this system. Johannes et al. [40] proposed an image processing algorithm combining candidate hot-spot detection with statistical inference methods and then developed a smartphone app for the identification of disease images of wheat septoria, rust, and tan spot. Based on DMIL and FCN, Lu et al. [28] proposed an in-field wheat disease identification and localization model called the Multiple Instance Learning-Based Wheat Disease Diagnosis System (DMIL-WDDS), and then they packed this model into a mobile app. In further studies, the parameters of the YOLOv5s-based disease identification model based on the disease images acquired at all the growth stages can be optimized, or other network models can be trained to improve the image identification accuracy and generalization ability of the built model, and then an easy-to-use computer system or app can be developed based on the built model.

## 4. Materials and Methods

**Figure 1:**
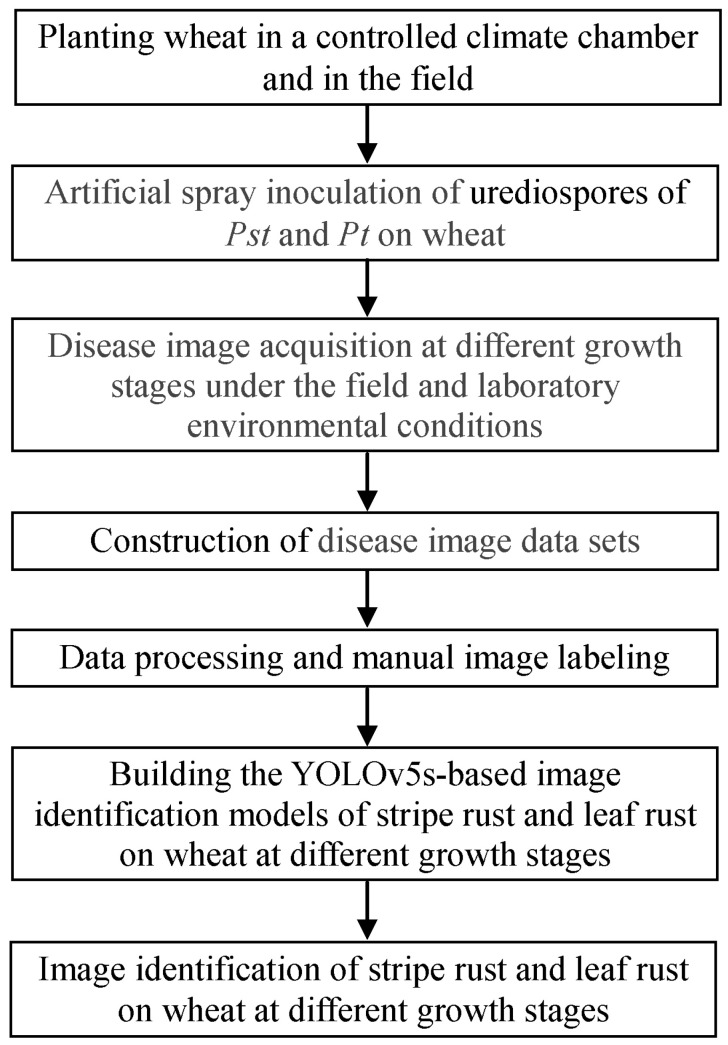
The workflow for building the YOLOv5s-based image identification models of wheat stripe rust and wheat leaf rust in the different growth stages.

In this study, YOLOv5s-based image identification models of wheat stripe rust and wheat leaf rust in the different growth stages were developed according to the workflow shown in Figure 1.

### 4.1. Planting Wheat and Artificial Spray Inoculation of Urediospores of Pst and Pt

The wheat planting was conducted in a controlled climate chamber (12–15 °C, 50–70% relative humidity, and 12 h/12 h light/dark cycle) in the Laboratory of Macro-Phytopathology, China Agricultural University, Beijing, China, a field at the Shangzhuang Experimental Station of China Agricultural University, Haidian District, Beijing, China, and a field at the Gangu Testing Station at the Institute of Plant Protection, Gansu Academy of Agricultural Sciences, Gangu, Gansu, China, using the methods previously described by Wang et al. [18]. In the controlled climate chamber, a wheat variety Mingxian 169 (highly susceptible to *Pst* and *Pt*) was used; at the Shangzhuang Experimental Station, wheat varieties Zhongmai 175 (highly resistant to *Pst* and moderately susceptible to *Pt*), Beijing 0045 (moderately resistant to *Pst* and *Pt*), Nongda 211 (moderately resistant to *Pst* and *Pt*), and Mingxian 169 were used; and at the Gangu Testing Station, wheat varieties Longjian 9825 (highly resistant to *Pst*), Tianxuan 66 (moderately resistant to *Pst*), and Longjian 9822 (moderately susceptible to *Pst*) were used. The inoculation operations of *Pst* and *Pt* were carried out using artificial spray inoculation methods. The pathogens (*Pst* and *Pt*) used for inoculation were multiplied under controlled climate chamber environments.

#### 4.1.1. Planting Wheat and Artificial Spray Inoculation of Urediospores of *Pst* and *Pt* in the Controlled Climate Chamber

In each pot with a diameter of 10 cm and a height of 10 cm, nursery soil containing organic matter was used as the substrate, and 15 seeds of Mingxian 169 were sown. After sowing, the pots were placed into the controlled climate chamber under the conditions described above. When the first leaves of the wheat seedlings of Mingxian 169 fully expanded, pathogen inoculation operations were carried out using the artificial spray inoculation method described by Cheng et al. [41]. Before inoculation, the inoculation operation environment, inoculation operation table, and inoculation tools were disinfected with a 75% ethanol solution. The emerging spear leaves of the wheat seedlings were cut off to retain only the first fully expanded leaves. The treated wheat seedlings were placed into an inoculation box with a small amount of water. To make the wheat seedlings more conducive to pathogen infection, the seedlings were sprayed evenly using a sprayer with clear water and were then gently washed by hand to remove the wax and pubescences on the surface of the leaves. The urediospore suspensions of *Pst* and *Pt* were made with 0.05% Tween 20 solution. The urediospore suspension of *Pst* or *Pt* was evenly sprayed onto the leaves of wheat seedlings in the inoculation box, and then the inoculation box was covered with its lid after the inside of the lid was sprayed with an appropriate amount of clear water. Subsequently, the inoculation box with the inoculated wheat seedlings was gently moved into the controlled climate chamber. After 24 h, each pot with the inoculated wheat seedlings was taken out of the inoculation box and was then covered with a transparent and clean glass cylinder that was wrapped with two layers of sterile cotton gauze on the top side. Finally, all the inoculated wheat seedlings were moved into the controlled climate chamber for incubation, and when appropriate, the seedlings were watered, and their emerging spear leaves were cut off.

#### 4.1.2. Planting Wheat and Artificial Spray Inoculation of Urediospores of *Pst* and *Pt* in the Field

At the Shangzhuang Experimental Station, wheat seeds were sown in October 2020, and October 2021. In each year, 21 experimental plots (3 × 4 m per plot) were set up. In 2020, Beijing 0045, Mingxian 169, and Zhongmai 175 were planted in the same order in each plot, and Nongda 211 was planted in protective belts between the plots. In 2021, Beijing 0045, Mingxian 169, and Nongda 211 were planted in the same order in each plot, and Zhongmai 175 was planted in protective belts between the plots. In April 2021, and April 2022, inoculation operations of *Pst* and *Pt* were carried out in the field using the artificial spray inoculation method described by Wang et al. [42]. In each year, *Pst* and *Pt* were inoculated in nine plots, and the remaining three plots were used as controls. In 2021, the urediospore suspensions at three concentrations of 300 mg/L, 200 mg/L, and 100 mg/L were used for the artificial spray inoculation of *Pst* and *Pt*. In 2022, the urediospore suspensions at three concentrations of 600 mg/L, 400 mg/L, and 200 mg/L were used for the artificial spray inoculation of *Pst* and *Pt*. The urediospore suspensions at each concentration were used to inoculate the wheat seedlings in three plots. Before inoculation, the wheat seedlings of a plot to be inoculated were sprayed evenly using a sprayer with clear water and were gently washed by hand to remove the wax and pubescences on the surface of the leaves. Subsequently, the treated wheat seedlings were evenly sprayed with the prepared urediospore suspension and were immediately covered with a plastic film sprayed with water droplets. To moisturize the inoculated wheat seedlings in the plot, the edges of the plastic film were covered with earth. Between 8:00 and 9:00 (Beijing Time) the next day, all of the inoculated plots were unveiled.

At the Gangu Testing Station, nine experimental plots (4.5 × 4.6 m per plot) were set up, and wheat seeds were sown with one wheat variety per plot in October 2020. Each of the wheat varieties Longjian 9825, Tianxuan 66, and Longjian 9822 was planted in three plots, respectively, with a cluster of Mingxian 169 planted in the center of each plot and Longjian 9825 planted in protective belts between the plots. In March 2021, the wheat seedlings of Mingxian 169 planted in the center of each plot were inoculated with a *Pst* urediospore suspension at a concentration of 1500 mg/L. After spraying the urediospore suspension, the wheat seedlings of Mingxian 169 were covered with a plastic bag, and then the edges of the plastic bag were covered with earth to moisturize the inoculated wheat seedlings. Between 8:00 and 9:00 (Beijing Time) the next day, all the seedlings were unveiled.

### 4.2. Acquisition of Disease Images and Construction of Disease Image Data Sets

In six growth stages of wheat, including the seedling growth stage, stem elongation stage, booting stage, inflorescence emergence stage, anthesis stage, and milk development stage, the acquisition of images of wheat stripe rust and wheat leaf rust was conducted. A Nikon D700 digital camera (Nikon Corp., Tokyo, Japan) and a HUAWEI P30 smartphone were used to acquire the disease images, and the sizes of the corresponding acquired disease images in jpeg format were 4256 × 2832 pixels and 3648 × 2736 pixels, respectively.

**Figure 2:**
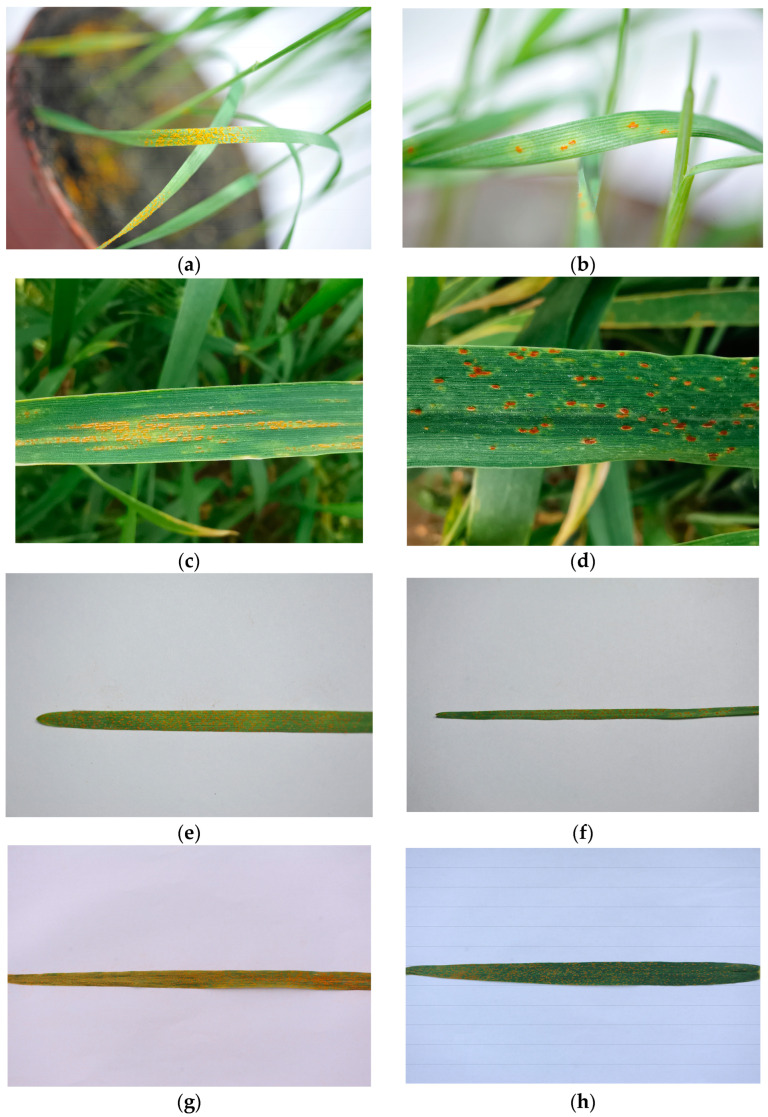
Images of wheat stripe rust and wheat leaf rust with complex backgrounds and single backgrounds acquired in an indoor environment and in the field. (**a**,**b**): Images of wheat stripe rust and wheat leaf rust, respectively, with complex backgrounds acquired in an indoor environment; (**c**,**d**): Images of wheat stripe rust and wheat leaf rust, respectively, with complex backgrounds acquired in the field; (**e**,**f**): Images of wheat stripe rust and wheat leaf rust, respectively, with single backgrounds acquired in an indoor environment; (**g**,**h**): Images of wheat stripe rust and wheat leaf rust, respectively, with single backgrounds acquired in the field.

In the seedling stage, images of wheat stripe rust and wheat leaf rust with complex backgrounds and single backgrounds were taken using a multi-angle image shooting method under different light intensity conditions in the indoor environment. To acquire the disease images with complex backgrounds, two large sheets of white paper with the size of 1 × 1 m were used as the wall background and the desktop background; a 10 × 10 cm white foam board was placed on the white paper covering the desktop, and the diseased wheat seedlings in the pot to be photographed were placed on the foam board; two table lamps were placed on the left and right sides of the seedlings to illuminate the diseased wheat leaves; and different light intensities were obtained by adjusting the positions and heights of the lamps when taking the disease images. In the stem elongation stage, booting stage, inflorescence emergence stage, anthesis stage, and milk development stage, the images of wheat stripe rust and wheat leaf rust with complex backgrounds and single backgrounds were taken from multiple angles in multiple time periods in the field environment. To acquire the disease images with single backgrounds in the indoor environment and in the field, single leaves with typical symptoms of wheat stripe rust or wheat leaf rust at different severity levels were collected from the diseased wheat seedlings, then each diseased single leaf was fully expanded as flat as possible on a sheet of A4 white paper. Subsequently, the images of the diseased single leaves were taken. The specific acquisition information on the images of wheat stripe rust and wheat leaf rust is shown in Table 8. The examples of the images of wheat stripe rust and wheat leaf rust with complex backgrounds and single backgrounds, acquired in the indoor environment and in the field, are shown in Figure 2.

In this study, a total of 15,722 images of wheat stripe rust and wheat leaf rust were acquired. Based on disease images with typical disease symptoms and different backgrounds acquired in an indoor environment and in the field, a total of seven image data sets in the seedling growth stage, stem elongation stage, booting stage, inflorescence emergence stage, anthesis stage, milk development stage, and all the growth stages, named Data Set 1, Data Set 2, Data Set 3, Data Set 4, Data Set 5, Data Set 6, and Data Set 7, respectively, were constructed. The image data set at all the growth stages, i.e., Data Set 7, was composed of all the disease images acquired in the different growth stages. Due to the short period available for disease image acquisition in the stem elongation stage of the wheat, the number of disease images acquired in the stem elongation stage is relatively small compared with the number of disease images acquired in the other growth stages. There are not many differences between the number of disease images contained in the disease image data sets in the seedling growth stage, booting stage, inflorescence emergence stage, anthesis stage, and milk development stage. The information on the seven constructed image data sets of wheat stripe rust and wheat leaf rust is shown in Table 9.

### 4.3. Configuration of the Data Analysis Operating Environment

Anaconda software (version 4.9.2) suitable for the system requirements of the computer used for data analysis was downloaded from the Anaconda official website (https://www.anaconda.com/download) and was then installed. After starting Anaconda, a new virtual environment of Python (version 3.8.12) named yolov5 was created for installing PyTorch. The “Win” and “R” keys on the keyboard were simultaneously pressed to launch the Run command window. The “cmd” was input into the Run command window, and then the “OK” button was clicked, and the command line window was opened. In the command line, the command “nvidia-smi” was input, and then the “Enter” key on the keyboard was pressed. In the pop-up interface, the computer graphics card information, including its driver version and supported CUDA (compute unified device architecture) version, could be checked. The corresponding supported CUDA version (version 10.2) was downloaded from the CUDA official website (https://developer.nvidia.com/cuda-toolkit-archive) and installed. Subsequently, the cuDNN version (version 8.1.0) corresponding to the CUDA was downloaded from the cuDNN official website (https://developer.nvidia.com/rdp/cudnn-archive) and installed. The created virtual environment yolov5 was activated, and then the corresponding version of PyTorch (version 1.10.0), i.e., the GPU version, was downloaded from the PyTorch official website (https://pytorch.org/) and installed.

From the GitHub website (https://github.com/ultralytics/yolov5), the source code of YOLOv5 was downloaded. In the Terminal window of PyCharm (version 2021.2.1) (a Python-integrated development environment), the command “pip install -r requirements.txt” was input to install the dependencies (packages) listed in the file requirements.txt in the root directory of the source code. After logging on to the official website to download the weight file of YOLOv5 (https://github.com/ultralytics/yolov5/releases), “Releases” was clicked, and then a web page appeared. In this web page, the YOLOv5 section was found, the weight file yolov5s.pt was downloaded from the Assets panel, and this weight file was placed into the root directory of the YOLOv5 source code.

The building, training, and testing of the YOLOv5s-based image identification models of stripe rust and leaf rust on wheat in different growth stages were conducted under the PyTorch framework using the CUDA parallel computing architecture. The configurations of the data analysis operating environment in detail are shown in Table 10.

### 4.4. Disease Image Labeling

The LabelImg software (version 1.8.6), an open-source data labeling tool, was used to label the images of wheat stripe rust and wheat leaf rust. Using the PyCharm software, the virtual environment yolov5 was launched. In the Terminal window, the command “pip install labelimg” was input to install the LabelImg software. Then, the command “labelimg” was input in the Terminal window to open the user interface of the LabelImg software. After the user interface of LabelImg was opened, the file folder where the wheat disease images to be labeled were located was selected by clicking the “Open Dir” button, and by clicking the “Change Save Dir” button, the file folder was selected or a new file folder was created for saving the class labels of the labeled wheat disease images. Then, the seventh option from top to bottom on the left side of the user interface was switched to YOLO. Subsequently, the “View” menu was clicked to select the following options: Auto Save mode (used to automatically save the class label of the labeled image when switching to the next unlabeled image), Display Labels (used to display the bounding boxes and class labels), and Advanced Mode (used to set the labeling crosshair to always appear on the user interface), and the disease images were manually labeled one by one. The left mouse button was pressed and dragged to create a bounding box to select the target to be labeled, and the class of the target was manually set to “TX” (referring to wheat stripe rust) or “YX” (referring to wheat leaf rust). The hotkeys used during labeling and their functions are as follows: “W” (used to create a bounding box), “A” (used to switch to the previous image), “D” (used to switch to the next image), and “Delete” (used to delete the selected bounding box). Each hotkey was used individually, and it was not necessary to press any other hotkeys simultaneously. If deleting a bounding box was required, the “Edit RectBox” button, the ninth option from top to bottom on the left side of the user interface of LabelImg, was clicked, then the bounding box to be deleted was selected*, and* subsequently, the “Delete” hotkey was pressed, and the bounding box was deleted. After labeling the wheat disease images, corresponding .txt label files were generated containing the target information, including the class, x_center (i.e., the normalized X coordinate of the center point of the bounding box of the target), y_center (i.e., the normalized Y coordinate of the center point of the bounding box of the target), width (i.e., the normalized width of the bounding box of the target), and height (i.e., the normalized height of the bounding box of the target).

### 4.5. Construction of the Training, Validation, and Testing Sets of the Disease Images

Each disease image in the image data sets in the seedling growth stage, stem elongation stage, booting stage, inflorescence emergence stage, anthesis stage, and milk development stage was labeled using LabelImg software, and according to the growth stages, the generated .txt label files were saved in the individual file folders corresponding to the growth stages. All the disease images in the image data sets were original, without any preprocessing. Based on the OS, Shutil, Random, and Tqdm libraries, using the Python programming language, both the disease images in the image data set at each growth stage and the .txt label files generated after the labeling of the corresponding images were divided into training, validation, and testing sets according to a ratio of 8:1:1. The training, validation, and testing sets of the disease images and the corresponding .txt label files for all the growth stages were composed of the images in the training, validation, and testing sets, and the corresponding .txt label files in the seedling growth stage, stem elongation stage, booting stage, inflorescence emergence stage, anthesis stage, and milk development stage. The number of disease images contained in the training, validation, and testing sets constructed using the images acquired during the different growth stages of the wheat is shown in Table 11.

### 4.6. Disease Image Identification Model Training and Testing

In the file folder named data in the root directory of the YOLOv5 source code, a file named YOLO.yaml was created. In the file YOLO.yaml, the “path” was followed by the saved path of the disease images for an individual growth stage or all the growth stages; “train” was followed by the path of the training set of the disease images; “val” was followed by the path of the validation set of the disease images; “test” was followed by the path of the testing set of the disease images; “nc” was followed by the number of classes, and in this study, the number of classes was “2”; “names” was followed by the class names, and in this study, the class names were “TX” and “YX”, referring to wheat stripe rust and wheat leaf rust, respectively. When training the YOLOv5s-based models for image identification of stripe rust and leaf rust on wheat in the seedling growth stage, stem elongation stage, booting stage, inflorescence emergence stage, anthesis stage, milk development stage, and all the growth stages, the paths following the “path”, “train”, “val”, and “test” in the file YOLO.yaml were changed according to the saved paths of the disease images and the corresponding .txt label files of the image data sets.

The file yolov5s.yaml, in the file folder named models in the root directory of the YOLOv5 source code was opened, and then the number following “nc” in this file was changed to “2”. The file hyp.scratch.yaml, located in the subfolder hyps in the file folder data, contains various hyperparameters, including learning rate, momentum, and weight decay. The hyperparameter values can be modified according to the model training requirements to optimize the model.

In this study, based on the training sets of wheat stripe rust and wheat leaf rust in the seedling growth stage, stem elongation stage, booting stage, inflorescence emergence stage, anthesis stage, milk development stage, and all the growth stages, the YOLOv5s network models were trained by running the file train.py located in the root directory of the YOLOv5 source code for building the image identification models of stripe rust and leaf rust on wheat in the seedling growth stage, stem elongation stage, booting stage, inflorescence emergence stage, anthesis stage, milk development stage, and all the growth stages. During the model training based on the training set of the disease images acquired during an individual growth stage or all the growth stages, the corresponding validation set was used, and the model weight file best.pt for the model with the best performance was saved. Then, the file best.pt was used to implement the tests of the built image identification model based on the testing sets by running the test.py file in the root directory of the YOLOv5 source code. In this study, because of the limitations of the hardware configuration conditions, the parameters for the training of the YOLOv5s-based image identification models of wheat stripe rust and wheat leaf rust in the different growth stages were set as shown in Table 12.

### 4.7. Evaluation Metrics of the Disease Image Identification Models

To evaluate the performance of the built YOLOv5-based image identification models of wheat stripe rust and wheat leaf rust for the identification of the images of individual diseases at the testing sets, Precision, Recall, F1 Score, and AP were used as the evaluation metrics. To evaluate the performance of the built YOLOv5-based image identification models for the identification of the images of wheat stripe rust and wheat leaf rust at the testing sets, mean F1 Score and mAP were used as the evaluation metrics.

Precision is used to describe the proportion of actual positive samples in the samples identified as positive samples in the final identification results, which can be calculated using Formula (1).

(1) $$\mathrm{Precision}=\frac{\mathrm{TP}}{\mathrm{TP}+\mathrm{FP}}\times 100\%$$

Recall is used to describe the proportion of identified positive samples in the samples that are actually positive, which can be calculated using Formula (2).

(2) $$\mathrm{Recall}=\frac{\mathrm{TP}}{\mathrm{TP}+\mathrm{FN}}\times 100\%$$

F1 Score is the harmonic mean of Precision and Recall, which can be calculated according to Formula (3).

(3) $$F1\;\mathrm{Score}=\frac{2\times \mathrm{Precision}\times \mathrm{Recall}}{\mathrm{Precision}+\mathrm{Recall}}$$

AP is used to describe the area below the Precision–Recall curve with Recall as the abscissa and Precision as the ordinate, which can be calculated according to Formula (4).

(4) $$\mathrm{AP}=\int_{0}^{1}\mathrm{Precision}\left(\mathrm{Recall}\right)d\mathrm{Recall}$$

Mean F1 Score is used to describe the mean of the values of the F1 Score for all the classes, which can be calculated using Formula (5).

(5) $$\mathrm{mean}\;F1\;\mathrm{Score}=\frac{\sum_{i=1}^{N}{F1\;\mathrm{Score}}_{i}}{N}$$

mAP is used to describe the mean of AP of all the classes, which can be calculated using Formula (6).

(6) $$\mathrm{mAP}=\frac{\sum_{i=1}^{N}{\mathrm{AP}}_{i}}{N}$$

In the above formulas, TP (true positive) represents the number of positive samples identified as positive samples; FP (false positive) represents the number of negative samples identified as positive samples; FN (false negative) represents the number of positive samples identified as negative samples; *N* denotes the number of the target classes; and *i* denotes the *i*th class.

## 5. Conclusions

In this study, based on the YOLOv5s model, the image identification models of stripe rust and leaf rust on wheat in the different individual growth stages and all the growth stages were developed, and by using the built YOLOv5s-based image identification models, the identification of the disease images in the testing sets of wheat stripe rust and wheat leaf rust in the different growth stages was conducted. The results showed that, for the YOLOv5s-based image identification model based on the training set of wheat stripe rust and wheat leaf rust in an individual growth stage, there were relatively great differences between the identification performances of the model on the testing sets for the different individual growth stages. Except for the YOLOv5s-based image identification model based on the training set of wheat stripe rust and wheat leaf rust in the milk development stage, the other YOLOv5s-based image identification models based on the training sets of wheat stripe rust and wheat leaf rust in the individual growth stages had the best identification performances on the testing sets in the same individual growth stages at which the images in the training sets used for modeling were acquired, and they were not suitable for identifying the images of wheat stripe rust and wheat leaf rust in the testing sets of the other individual growth stages. The YOLOv5s-based disease image identification model based on the training set at all the growth stages was used to identify the disease images of wheat stripe rust and wheat leaf rust in the testing sets in the seedling stage, stem elongation stage, booting stage, inflorescence emergence stage, anthesis stage, milk development stage, and all the growth stages, and acceptable identification performances were achieved on all the testing sets of the different individual growth stages and all the growth stages. The model had the best identification performance on the images of wheat stripe rust and wheat leaf rust in the testing set in the seedling stage, the identification performance of the model on the images of wheat stripe rust and wheat leaf rust in the testing set in the inflorescence emergence stage ranked second, and the model had the worst identification performance on the images of wheat stripe rust and wheat leaf rust in the testing set in the stem elongation stage. Furthermore, the results indicated that the growth stages of wheat had a great influence on the image-based identification of wheat stripe rust and wheat leaf rust using image processing technology. This study provided a reference for further building plant disease image identification models.

## Figures and Tables

**Table 1:** Disease identification results for the testing sets in the different individual growth stages using the YOLOv5s-based model based on the training set of wheat stripe rust and wheat leaf rust during the seedling stage.

Testing Set	Wheat Disease	Precision	Recall	F1 Score	AP	Mean F1 Score	mAP
Testing set at the seedling growth stage	Wheat stripe rust	81.30%	85.80%	83.49%	89.60%	78.12%	82.45%
	Wheat leaf rust	76.20%	69.60%	72.75%	75.30%		
Testing set at the stem elongation stage	Wheat stripe rust	57.40%	10.20%	17.32%	33.20%	31.95%	37.50%
	Wheat leaf rust	44.30%	49.10%	46.58%	41.80%		
Testing set at the booting stage	Wheat stripe rust	62.30%	28.20%	38.83%	42.80%	41.52%	38.25%
	Wheat leaf rust	42.30%	46.30%	44.21%	33.70%		
Testing set at the inflorescence emergence stage	Wheat stripe rust	63.70%	22.30%	33.04%	37.50%	30.81%	28.20%
	Wheat leaf rust	21.10%	44.30%	28.59%	18.90%		
Testing set at the anthesis stage	Wheat stripe rust	34.00%	18.00%	23.54%	23.30%	34.49%	32.95%
	Wheat leaf rust	33.50%	70.60%	42.60%	45.44%		
Testing set at the milk development stage	Wheat stripe rust	47.90%	12.50%	28.10%	19.83%	29.80%	33.95%
	Wheat leaf rust	28.80%	64.30%	39.80%	39.78%		

Note: In this table and the tables below, the seedling growth stage, stem elongation stage, booting stage, inflorescence emergence stage, anthesis stage, and milk development stage refer to the stages of Z12–Z14, Z30–Z39, Z40–Z49, Z50–Z59, Z60–Z69, and Z70–Z77, respectively, according to the decimal code for growth stages of cereals determined by Zadoks et al. [36].

**Table 2:** Disease identification results for the testing sets in the different individual growth stages using the YOLOv5s-based model based on the training set of wheat stripe rust and wheat leaf rust in the stem elongation stage.

Testing Set	Wheat Disease	Precision	Recall	F1 Score	AP	Mean F1 Score	mAP
Testing set at the seedling growth stage	Wheat stripe rust	62.30%	57.80%	59.97%	58.30%	56.86%	54.55%
	Wheat leaf rust	53.70%	53.80%	53.75%	50.80%		
Testing set at the stem elongation stage	Wheat stripe rust	68.80%	55.40%	61.38%	63.20%	63.08%	63.55%
	Wheat leaf rust	63.80%	65.80%	64.78%	63.90%		
Testing set at the booting stage	Wheat stripe rust	49.70%	49.80%	49.75%	48.80%	45.75%	42.15%
	Wheat leaf rust	36.20%	49.30%	41.75%	35.50%		
Testing set at the inflorescence emergence stage	Wheat stripe rust	45.70%	36.80%	40.77%	38.90%	38.39%	32.95%
	Wheat leaf rust	33.60%	38.80%	36.01%	27.00%		
Testing set at the anthesis stage	Wheat stripe rust	32.90%	29.80%	31.27%	26.10%	37.19%	30.35%
	Wheat leaf rust	39.00%	48.20%	43.11%	34.60%		
Testing set at the milk development stage	Wheat stripe rust	21.90%	26.00%	23.77%	15.70%	34.09%	27.75%
	Wheat leaf rust	38.70%	52.10%	44.41%	39.80%		

**Table 3:** Disease identification results for the testing sets in the different individual growth stages using the YOLOv5s-based model based on the training set of wheat stripe rust and wheat leaf rust in the booting stage.

Testing Set	Wheat Disease	Precision	Recall	F1 Score	AP	Mean F1 Score	mAP
Testing set at the seedling growth stage	Wheat stripe rust	75.80%	54.60%	63.48%	66.20%	55.92%	55.65%
	Wheat leaf rust	53.70%	44.00%	48.37%	45.10%		
Testing set at the stem elongation stage	Wheat stripe rust	75.20%	29.00%	41.86%	51.80%	49.39%	53.90%
	Wheat leaf rust	63.30%	51.70%	56.91%	56.00%		
Testing set at the booting stage	Wheat stripe rust	68.80%	65.20%	66.95%	72.60%	62.45%	64.85%
	Wheat leaf rust	56.30%	59.70%	57.95%	57.10%		
Testing set at the inflorescence emergence stage	Wheat stripe rust	77.70%	58.20%	66.55%	70.10%	61.67%	62.60%
	Wheat leaf rust	56.40%	57.20%	56.80%	55.10%		
Testing set at the anthesis stage	Wheat stripe rust	60.70%	54.20%	57.27%	56.90%	57.66%	56.25%
	Wheat leaf rust	52.70%	64.60%	58.05%	55.60%		
Testing set at the milk development stage	Wheat stripe rust	46.60%	51.50%	48.93%	46.80%	49.96%	47.85%
	Wheat leaf rust	53.90%	48.40%	51.00%	48.90%		

**Table 4:** Disease identification results for the testing sets in the different individual growth stages using the YOLOv5s-based model based on the training set of wheat stripe rust and wheat leaf rust in the inflorescence emergence stage.

Testing Set	Wheat Disease	Precision	Recall	F1 Score	AP	Mean F1 Score	mAP
Testing set at the seedling growth stage	Wheat stripe rust	53.40%	54.10%	53.75%	53.20%	46.33%	43.10%
	Wheat leaf rust	49.40%	32.10%	38.91%	33.00%		
Testing set at the stem elongation stage	Wheat stripe rust	58.00%	20.30%	30.07%	38.90%	39.39%	44.15%
	Wheat leaf rust	65.70%	38.70%	48.71%	49.40%		
Testing set at the booting stage	Wheat stripe rust	56.80%	63.00%	59.74%	62.30%	54.84%	53.70%
	Wheat leaf rust	55.80%	45.20%	49.94%	45.10%		
Testing set at the inflorescence emergence stage	Wheat stripe rust	74.70%	75.90%	75.30%	79.90%	69.06%	71.45%
	Wheat leaf rust	67.30%	58.90%	62.82%	63.00%		
Testing set at the anthesis stage	Wheat stripe rust	59.40%	62.50%	60.91%	62.50%	60.46%	60.35%
	Wheat leaf rust	61.60%	58.50%	60.01%	58.20%		
Testing set at the milk development stage	Wheat stripe rust	53.80%	62.00%	57.61%	54.60%	54.26%	49.95%
	Wheat leaf rust	56.40%	46.40%	50.91%	45.30%		

**Table 5:** Disease identification results for the testing sets in the different individual growth stages using the YOLOv5s-based model based on the training set of wheat stripe rust and wheat leaf rust in the anthesis stage.

Testing Set	Wheat Disease	Precision	Recall	F1 Score	AP	Mean F1 Score	mAP
Testing set at the seedling growth stage	Wheat stripe rust	69.30%	35.30%	46.77%	54.60%	40.33%	41.50%
	Wheat leaf rust	44.40%	27.40%	33.89%	28.40%		
Testing set at the stem elongation stage	Wheat stripe rust	75.30%	14.50%	24.32%	44.40%	31.19%	42.50%
	Wheat leaf rust	56.10%	28.80%	38.06%	40.60%		
Testing set at the booting stage	Wheat stripe rust	69.00%	59.70%	64.01%	64.60%	54.48%	52.40%
	Wheat leaf rust	46.70%	43.30%	44.94%	40.20%		
Testing set at the inflorescence emergence stage	Wheat stripe rust	76.80%	60.40%	67.62%	70.60%	63.56%	63.80%
	Wheat leaf rust	62.20%	57.00%	59.49%	57.00%		
Testing set at the anthesis stage	Wheat stripe rust	68.50%	71.80%	70.11%	72.70%	72.11%	75.30%
	Wheat leaf rust	71.60%	76.80%	74.11%	77.90%		
Testing set at the milk development stage	Wheat stripe rust	64.40%	57.90%	60.98%	62.30%	59.97%	59.95%
	Wheat leaf rust	62.50%	55.80%	58.96%	57.60%		

**Table 6:** Disease identification results for the testing sets in the different individual growth stages using the YOLOv5s-based model constructed based on the training set of wheat stripe rust and wheat leaf rust in the milk development stage.

Testing Set	Wheat Disease	Precision	Recall	F1 Score	AP	Mean F1 Score	mAP
Testing set at the seedling growth stage	Wheat stripe rust	49.20%	28.80%	36.33%	41.30%	34.62%	35.60%
	Wheat leaf rust	47.40%	25.20%	32.91%	29.90%		
Testing set at the stem elongation stage	Wheat stripe rust	52.60%	11.10%	18.33%	32.30%	29.75%	37.75%
	Wheat leaf rust	56.70%	32.30%	41.16%	43.20%		
Testing set at the booting stage	Wheat stripe rust	60.50%	51.80%	55.81%	59.10%	52.37%	50.05%
	Wheat leaf rust	51.00%	47.00%	48.92%	41.00%		
Testing set at the inflorescence emergence stage	Wheat stripe rust	64.30%	61.80%	63.03%	64.50%	55.40%	54.55%
	Wheat leaf rust	42.70%	54.20%	47.77%	44.60%		
Testing set at the anthesis stage	Wheat stripe rust	63.10%	62.90%	63.00%	63.10%	65.29%	65.85%
	Wheat leaf rust	66.50%	68.70%	67.58%	68.60%		
Testing set at the milk development stage	Wheat stripe rust	63.90%	65.10%	64.49%	68.00%	65.00%	67.20%
	Wheat leaf rust	62.60%	68.70%	65.51%	66.40%		

**Table 7:** Disease identification results for the testing sets in the different individual growth stages and all the growth stages using the YOLOv5s-based model constructed based on the training set of wheat stripe rust and wheat leaf rust in all the growth stages.

Testing Set	Wheat Disease	Precision	Recall	F1 Score	AP	Mean F1 Score	mAP
Testing set at the seedling growth stage	Wheat stripe rust	84.40%	84.40%	84.40%	89.00%	79.98%	82.80%
	Wheat leaf rust	75.60%	75.50%	75.55%	76.60%		
Testing set at the stem elongation stage	Wheat stripe rust	70.00%	56.80%	62.71%	66.80%	64.06%	66.55%
	Wheat leaf rust	65.80%	65.00%	65.40%	66.30%		
Testing set at the booting stage	Wheat stripe rust	74.70%	69.20%	71.84%	74.50%	65.94%	68.05%
	Wheat leaf rust	61.10%	59.00%	60.03%	61.60%		
Testing set at the inflorescence emergence stage	Wheat stripe rust	78.60%	75.20%	76.86%	80.20%	71.48%	74.90%
	Wheat leaf rust	71.30%	61.60%	66.10%	69.60%		
Testing set at the anthesis stage	Wheat stripe rust	69.00%	74.20%	71.51%	74.70%	70.89%	74.40%
	Wheat leaf rust	68.90%	71.70%	70.27%	74.10%		
Testing set at the milk development stage	Wheat stripe rust	69.00%	68.40%	68.70%	70.40%	67.11%	68.55%
	Wheat leaf rust	62.70%	68.60%	65.52%	66.70%		
Testing set at all the growth stages	Wheat stripe rust	73.60%	67.60%	70.47%	74.60%	68.95%	71.95%
	Wheat leaf rust	68.60%	66.30%	67.43%	69.30%		

**Table 8:** The specific acquisition information on the images of wheat stripe rust and wheat leaf rust.

Wheat Disease	Wheat Growth Stage	Acquisition Date	Diseased Leaf Sampling Location
Wheat stripe rust	Seedling growth stage	October to December, 2021	The controlled climate chamber in the Laboratory of Macro-Phytopathology, China Agricultural University, Beijing, China
Wheat stripe rust	Other growth stages	April to June 2021, and April to May 2022	The Shangzhuang Experimental Station of China Agricultural University, Haidian District, Beijing, China, and the Gangu Testing Station at the Institute of Plant Protection, Gansu Academy of Agricultural Sciences, Gangu, Gansu, China
Wheat leaf rust	Seedling growth stage	April to July 2021, and April, July, September, and October, 2022	The controlled climate chamber in the Laboratory of Macro-Phytopathology, China Agricultural University, Beijing, China
Wheat leaf rust	Other growth stages	April to June 2021, and April to May 2022	The Shangzhuang Experimental Station of China Agricultural University, Haidian District, Beijing, China

**Table 9:** Quantities of disease images in the data sets constructed for the identification of wheat stripe rust and wheat leaf rust in the different growth stages.

Data Set	Wheat Growth Stage	The Number of the Images of Wheat Stripe Rust	The Number of the Images of Wheat Leaf Rust	The Total Number of Disease Images
Data Set 1	Seedling growth stage	1430	1430	2860
Data Set 2	Stem elongation stage	1187	1020	2207
Data Set 3	Booting stage	1261	1316	2577
Data Set 4	Inflorescence emergence stage	1386	1278	2664
Data Set 5	Anthesis stage	1379	1309	2688
Data Set 6	Milk development stage	1374	1352	2726
Data Set 7	All growth stages	8017	7705	15722

**Table 10:** Data analysis operating environment used in this study.

Item	Configuration
Operating system	Windows10
Memory	16 GB
Graphics processing unit (GPU)	NVIDIA Quadro P1000
Central processing unit (CPU)	Intel(R) Core(TM) i7-9700
PyCharm version	2021.2.1
Python version	3.8.12
PyTorch version	1.10.0
CUDA version	10.2
cuDNN version	8.1.0

**Table 11:** Quantities of disease images contained in the training, validation, and testing sets constructed using the images acquired during the different growth stages of wheat.

Wheat Growth Stage	The Number of Disease Images in the Training Set	The Number of Disease Images in the Validation Set	The Number of Disease Images in the Testing Set
Seedling growth stage	2288	286	286
Stem elongation stage	1765	221	221
Booting stage	2061	258	258
Inflorescence emergence stage	2130	267	267
Anthesis stage	2150	269	269
Milk development stage	2180	273	273
All growth stages	12574	1574	1574

**Table 12:** The set parameters for training the YOLOv5s-based models based on the training sets of wheat stripe rust and wheat leaf rust in the different growth stages.

Parameter	Value
Image size/pixel	640 × 640
Batch size	4
Learning rate	0.001
Epochs	80
Optimizer	Adam
Weight decay	0.0005
Momentum	0.937

## Data Availability

The data presented in this study are available on request from the corresponding author.
